# A Rare Presentation of a Rare Case: Acute Respiratory Failure in Swyer-James-Macleod Syndrome

**DOI:** 10.7759/cureus.13347

**Published:** 2021-02-15

**Authors:** Ashesha Mechineni, Balraj Singh, Rajapriya Manickam

**Affiliations:** 1 Internal Medicine, St. Joseph's University Medical Center, Paterson, USA; 2 Hematology/Oncology, St. Joseph's University Medical Center, Paterson, USA; 3 Pulmonary/Critical Care Medicine, St. Joseph's University Medical Center, Paterson, USA

**Keywords:** swyer-james syndrome, mechanical ventilation, swyer-james-macleod syndrome, hyperlucent lung, obliterative bronchiolitis, pulmonary artery hypoplasia, rare clinical entity

## Abstract

Swyer-James-Macleod syndrome (SJMS) is a rare clinical entity acquired during childhood due to a respiratory infection leading to bronchiolitis obliterans. This inciting event is hypothesized to cause structural and functional changes of the developing alveoli, terminal bronchioles, and the corresponding pulmonary vasculature, resulting in emphysematous changes and a matched ventilation-perfusion defect. We present a 67-year-old male patient with hypercapnic respiratory failure requiring invasive mechanical ventilation, who had typical features of SJMS undiagnosed before this admission. He was extubated successfully, discharged home, and continues to be stable at a 90-day follow-up period. This marks one of the rare accounts where a patient with SJMS was given ventilatory support emergently, and one of the oldest patients reported. SJMS is under-reported due to its indolent clinical course and misdiagnosed as some other pulmonary abnormality. The clinical course progression and prognosis are unclear and variable in many affected patients due to this condition's rarity.

## Introduction

Swyer-James-Macleod syndrome (SJMS) was named after the three physicians who described them simultaneously in the 1950s [1,2]. The clinical course of this rare entity is unclear, especially in elderly patients. We present an elderly male with co-morbidities and undiagnosed SJMS presenting with acute hypercapnic respiratory failure.

## Case presentation

**Figure 1:**
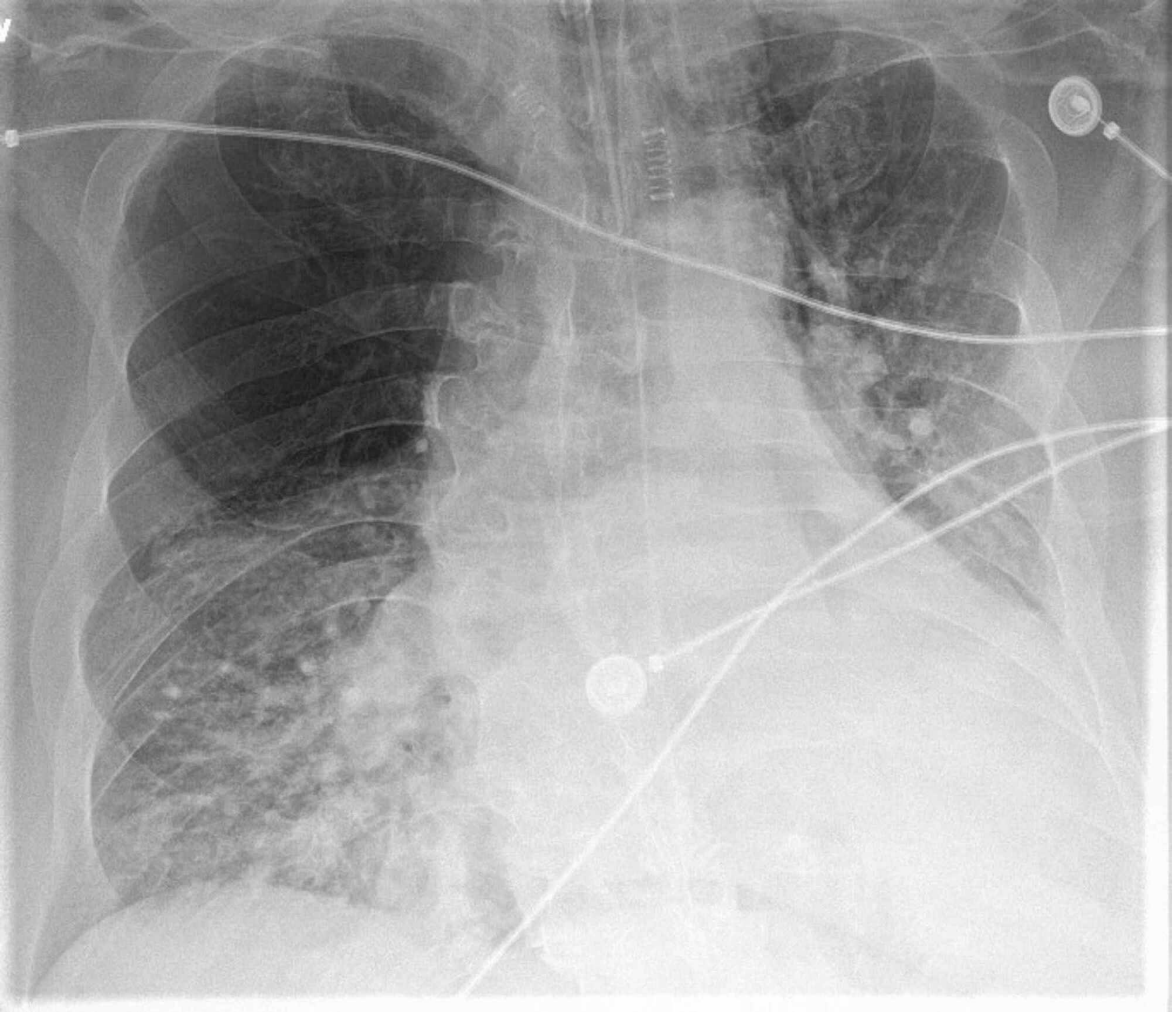
Chest radiograph of SJMS Posteroanterior view of portable chest radiograph of the patient on presentation showing the signature finding of SJMS, right upper lobe hyperlucency. SJMS - Swyer-James-Macleod syndrome

**Figure 2:**
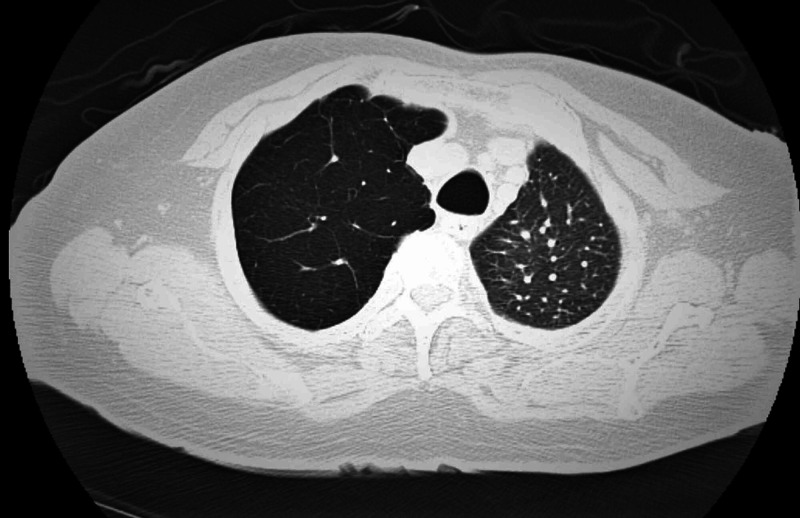
Axial view of computed tomography of the chest without contrast Computed tomography of the chest without contrast demonstrating right upper lobe air trapping and volume loss of left hemithorax.

**Figure 3:**
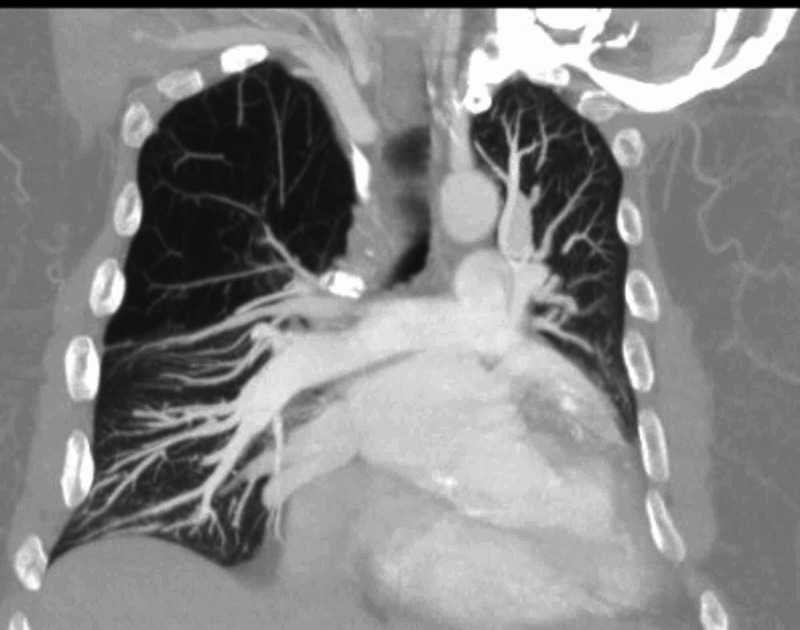
Coronal view of CT angiogram of chest CT angiography of the patient showing small-calibre pulmonary vasculature of right upper lobe consistent with SJMS with associated pulmonary changes mainly paucity of broncho-alveolar structures. CT - Computed tomography

A 67-year-old non-smoker male with a history of scoliosis, hypertension, chronic diastolic heart failure, and remote history of tobacco use approximately about 10-pack-years, quit more than 25 years ago, presented to St. Joseph's University Medical Center in New Jersey with a one-month history of progressively worsening exertional dyspnea, orthopnea, and dry cough. Vital signs revealed hypoxia on ambient air. Physical examination notable for tachypnea, mild scoliosis, and mild bilateral pedal edema. On auscultation, breath sounds were diminished on the right side anteriorly, mild crackles in bilateral bases, and no rhonchi or wheeze throughout lung fields. The chest radiograph showed hyperlucency of the right upper lobe (Figure 1) and bilateral lower lobe atelectasis. Arterial blood gas revealed acute hypercapnia and hypoxia. The patient was managed as an acute exacerbation of obstructive lung disease and acute exacerbation of diastolic heart failure. He failed bilevel positive airway pressure and required mechanical ventilation. Computed tomography (CT) without contrast (Figure 2) and CT angiography of the chest done during a prior hospitalization (Figure 3) revealed features typical of SJMS. Fiberoptic bronchoscopy and alpha-1 anti-trypsin levels were normal. An echocardiogram showed grade 1 diastolic dysfunction and no pulmonary hypertension. Though he had diastolic dysfunction, pulmonary edema was not significant alone to cause the respiratory comprise. We realized that underlying SJMS was an important factor causing hypercapnic respiratory failure. Based on the clinical picture, we managed him with nebulizer therapy including short-acting beta-agonist and inhaled corticosteroid. Ventilation strategies used were avoidance of high tidal volumes and high peak airway pressures with the successful outcome of extubation.

## Discussion

In SJMS, childhood respiratory infection causes inflammation and destruction of focal alveolar structures resulting in their underdevelopment, hypoperfusion, and significant air trapping [3]. The heterogeneous nature of resultant lung changes translates into a wide variety of clinical presentations. It is usually diagnosed in childhood with repeated pulmonary infections [4]. One study estimates the prevalence of the condition as 0.01% after review of 17,450 chest radiographs [5]. Most of the cases reported who were diagnosed as adult patients are below 60 years of age [6-9] compared to two reports of elderly patients who were 72 and 73 years at the time of diagnosis [7,10]. The classic triad for diagnosis is unilateral hyperlucent lung, diffusely decreased ventilation, and matching decreased perfusion in the affected lung [11]. Differential diagnoses are congenital lobar emphysema, pulmonary hypoplasia, bullous emphysema, and pulmonary embolism.

High-resolution CT (HRCT) is more sensitive in detecting the alveolar distortion and extent of involvement. CT angiography demonstrates hypoplasia of the pulmonary artery and its branches on the affected side. The narrowed attenuated arteries coursing through the radiolucent lung produce a "pruned‐tree" appearance. Ventilation perfusion scan and magnetic resonance angiography may be needed in selective cases [12]. Pulmonary function tests show a mixed obstructive-restrictive pattern. There are few reports where SJMS was misdiagnosed as a chronic obstructive pulmonary disease (COPD) [9], pulmonary embolism [8], and pneumothorax [13]. Conservative management that aims to improve airway clearance, prevent infections, and preserve pulmonary function is appropriate in most cases. Surgical treatment is reserved for patients with refractory infections [14].

## Conclusions

SJMS is an under-reported clinical syndrome in adults and especially elderly, frequently misdiagnosed and mistreated. The overall prognosis depends on the extent of the structural abnormality and the presence of other co-morbidities. It should be considered in cases with atypical emphysematous findings on chest radiograph.
